# Mobility Assessment of the Supraspinatus in a Porcine Cadaver Model Using a Sensor-Enhanced, Arthroscopic Grasper

**DOI:** 10.1007/s10439-020-02572-3

**Published:** 2020-08-13

**Authors:** Felix Porschke, Christoph Luecke, Thorsten Guehring, Christel Weiss, Stefan Studier-Fischer, Paul Alfred Gruetzner, Marc Schnetzke

**Affiliations:** 1 BG Trauma Center Ludwigshafen at Heidelberg University Hospital, Ludwig-Guttmann-Straße 13, 67071 Ludwigshafen, Germany; 2 Arcus Sportklinik, Rastatter Str. 17-19, 75179 Pforzheim, Germany; 3 Department of Medical Statistics, University Medicine Mannheim, Medical Faculty Mannheim of the University of Heidelberg, 68167 Mannheim, Germany; 4 German Joint Center, Atos Clinic, Bismarckstraße 9-15, 69115 Heidelberg, Germany

**Keywords:** Tendon, Rotator cuff, Arthroscopy, Biomechanics

## Abstract

Tendon mobility is highly relevant in rotator cuff surgery. Objective data about rotator cuff mobility is rare. Tendon mobility still needs to be evaluated subjectively by the surgeon. This study aims to establish a porcine animal model for mobility analysis of the supraspinatus. In this context, we introduce a sensor-enhanced, arthroscopic grasper (SEAG) suitable for objective intraoperative measurements of tendon mobility in clinical praxis. Tendon mobility of 15 fresh porcine cadaver shoulders with artificial rotator cuff tears was evaluated using the SEAG. Mobility characteristics (load–displacement curves, maximum load, stiffness) were studied and inter- and intraobserver agreement (intraclass correlation coefficient (ICC)) were tested. Factors with a potential adverse effect (plastic deformation and rigor mortis) were also evaluated. All shoulders showed characteristic reproducible load–displacement curves with a nonlinear part at the start, followed by a linear part. Mean maximum load was 28.6 N ± 12.5. Mean stiffness was 6.0 N/mm ± 2.6. We found substantial interobserver agreement (ICC 0.672) and nearly perfect intraobserver agreement (0.944) for maximum load measurement. Inter- (0.021) and intraobserver (0.774) agreement for stiffness was lower. Plastic deformation and rigor mortis were excluded. The animal model demonstrates reliable and *in vivo*-like measurements of tendon mobility. The SEAG is a reliable tool for tendon mobility assessment.

## Introduction

Rotator cuff injury is a frequent musculoskeletal disease and causes pain and functional impairment. Reconstruction of rotator cuff defects has become increasingly important due to advances in surgical techniques and the development of new implants. Despite such progress, a considerable percentage of patients still suffers from a re-tear after tendon reconstruction.^15^,^34^ Clinical and experimental research has focused on the insertion site (bone-implant and implant-tendon interface) of tendon reconstruction. The biomechanical properties of various repair techniques have been thoroughly investigated using animal or human cadaver models.

Due to the natural course of tendon retraction and remodeling, the intraoperative mobility of a torn tendon is also an important factor.^14^,^28^ Several radiological parameters have been identified as predictors for clinical outcome and re-tear rates.^26^,^33^ Tendon mobility could be an important link or even an explanation for worse outcomes in the presence of a retracted tendon^17^,^28^ or atrophy of the muscle^14^,^37^ in MRI.

In addition, tendon mobility is an important factor to consider in decisions about the best individual surgical procedure. Different surgical methods have been implemented to improve mobility and therefore reduce the strain of torn tendons in rotator cuff reconstruction.^1^,^18^,^25^ Tauro *et al*. introduced the arthroscopic interval slide procedure and reported favorable results in comparison to non-released shoulders.^36^ These results were reproduced by Lo and Burkhardt. They even advanced this procedure with additional posterior release.^25^

Recently, worse outcomes for patients with high tendon tension at time of reconstruction were found.^16^,^31^ Mobility might therefore prescribe the reconstruction technique.^5^,^19^

Despite the growing interest in tendon mobility, objective measurement methods for it are still lacking. Therefore, surgeons need to apply their experience to assess the tendon mobility correctly. Furthermore, no model exists for investigating rotator cuff tendon mobility. Several *in vitro* studies have focused on tensile properties of exemplary parts of the rotator cuff.^10^,^12^,^21^ However, the entire myotendinous unit has rarely been investigated.

The aim of this feasibility study was to establish an *in vivo*-like model for mobility analyses of the supraspinatus myotendinous unit. A second aim was to introduce a prototype of a sensor-enhanced, arthroscopic grasper (SEAG) for objective tendon mobility measurements which could be transferred to the clinical setting. The following design criteria were set to ensure practicability in clinical praxis: The SEAG must be portable, have similar dimensions to standard arthroscopic instruments, and be sterilizable. Further grasping and the release of the tendon stump should be fast and intuitive to avoid unnecessary extension of the operation. With regard to existing data, the SEAG must provide high accuracy and a measuring range between 0.5 and 100 N.^4^ This was achieved by connecting a commercial reusable arthroscopic grasper with self-releasing locking mechanism *via* a sterilizable custom-made aluminum fast adapter (Fa. Surgitaix) to an industrial force gauge. Furthermore, tendon mobility measurement using the SEAG must fulfill the criteria of reliability. We therefore analyzed force–displacement characteristics of 15 porcine supraspinatus tendons measured by the SEAG and evaluated inter- and intraobserver agreement.

## Materials and Methods

### Specimen Preparation

**Figure 1:**
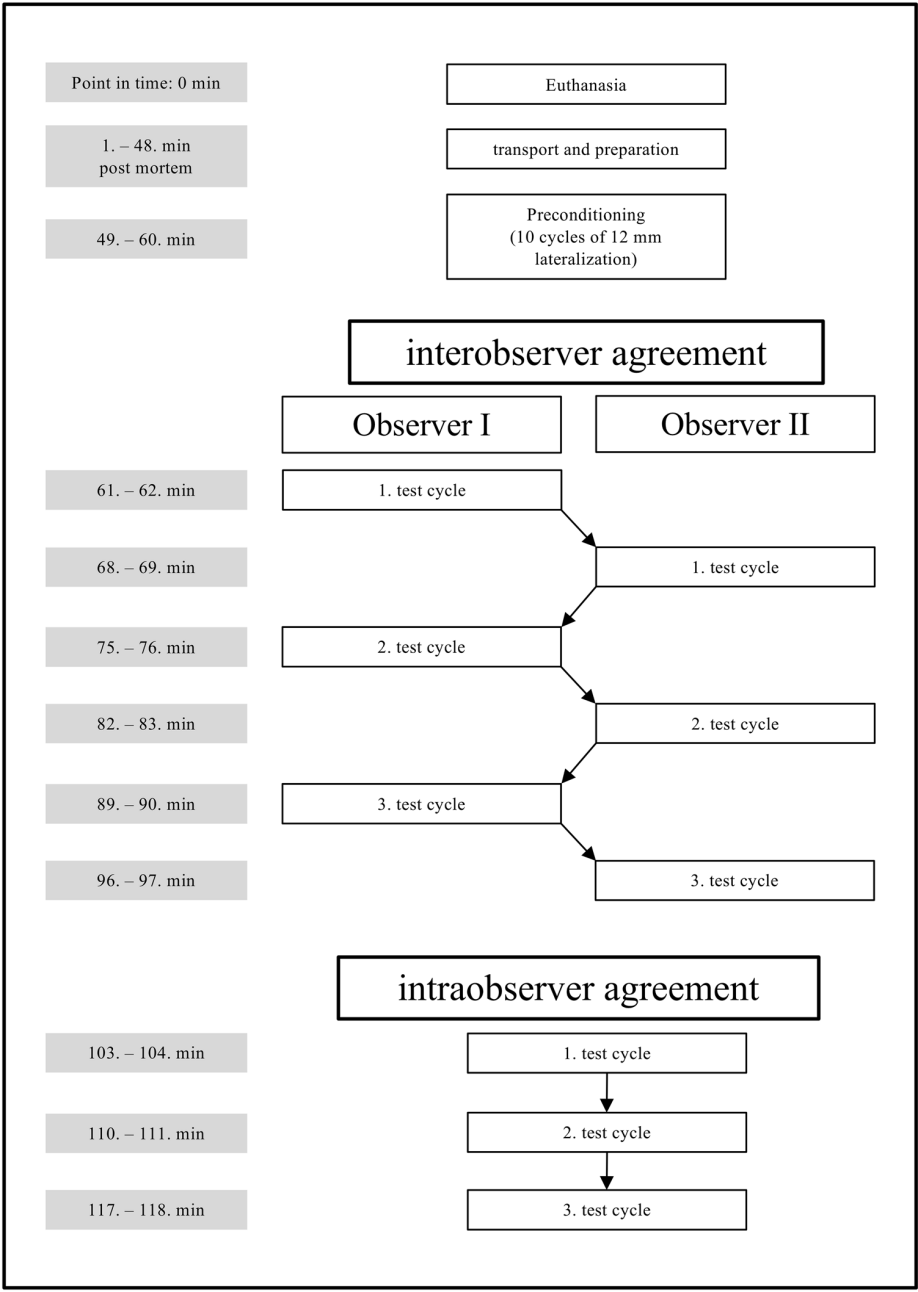
Test protocol with timetable.

15 porcine shoulders were harvested from 15 euthanized domestic pigs (*Sus scrofa forma domestica*). Due to their original purpose for the food industry no approval of an animal welfare organization was needed. Within 50 min postmortem, the shoulders were transferred to the laboratory and prepared. After cyclic preloading (10 min) the first measurements started 1 h postmortem. Harvesting, preparation, and all measurements were completed within 2 h postmortem using a rigorous schedule to guarantee *in vivo*-like tissue properties^38^ (Fig. 1).

The laboratory air-conditioning system kept the ambient temperature constant at 20 °C ± 0.5 and humidity at 45% ± 2.5.

Each shoulder was disarticulated from the thorax, by avoiding any damage to the subscapularis, supraspinatus, infraspinatus, and deltoid. The humerus was exarticulated in the elbow joint. Biceps and coracobrachialis muscles were resected distally at the height of the elbow joint.

The scapula was fixed using three bicortical screws (superior angle, inferior angle and glenoid neck) with the medial border perpendicular to the ground and the subscapularis facing a mounting plate to the testing station. Contact pressure between the mounting plate and subscapularis muscle was prevented by screw nuts used as spacers between the shoulders and the testing station. The humeral shaft was distally fixed at 30° glenohumeral abduction using another bicortical screw.

Skin and subcutaneous fat were removed. The deltoid muscle was detached from its insertion at the proximal humerus. The distal portion of the muscle was resected allowing access to the supraspinatus insertion, while the proximal part of the muscle was not touched to preserve the anatomic sliding layers.

### Defect Creation

The supraspinatus tendon was detached from its insertion site on the proximal humerus to mimic a full-thickness tear without retraction. The tendon width (distance between anterior and posterior tendon margin at the lateral tendon stump) and length of the entire myotendinous unit (from most medial insertion of supraspinatus muscle to lateral tendon stump) were obtained using a digital caliper.

### Testing Station

**Figure 2:**
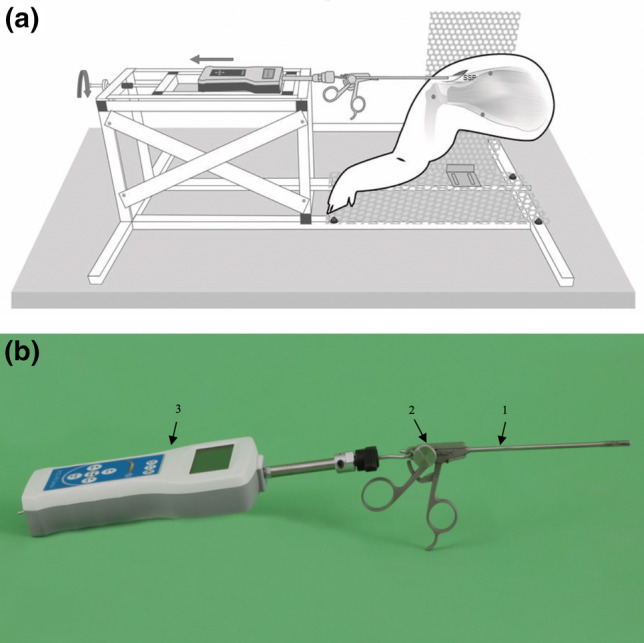
(a) Schematic illustration of the custom-designed testing station. The SEAG is aligned with the supraspinatus muscle (SSP) and grasps the distal tendon stump. The SEAG is lateralized in 1 mm steps *via* a slide by using an isometric screw (left). (b) Sensor enhanced arthroscopic grasper (SEAG): An arthroscopic grasper (1) is connected in series *via* a custom-made aluminum fast adapter (2) to a force gauge (3).

The custom-designed testing station was built from 23.5 mm aluminum rectangular tubes and perforated plates (Fig. 2a). A unique feature was a 317 mm × 157 mm slide for the SEAG, which enables lateralization of the SEAG in millimeter intervals using an isometric screw (M6 acc. DIN 13-1). Additional cross braces were used to prevent distortion of any part of the testing station.

### Sensor Enhanced Arthroscopic Grasper

For the SEAG, a commercial arthroscopic grasper (Rotator cuff grasper: 4.2 mm shaft diameter with traumatic jaws, and a self-releasing locking mechanism from Fa. Arthrex Inc.) was connected in series *via* a custom-made aluminum fast adapter (Fa. Surgitaix) to an industrial force gauge (PCE-FB 200, accuracy ± 0.1%, precision 0.05 N, measuring range 0.5–200 N, Fa. PCE Instruments) (Fig. 2b). The arthroscopic grasper and adapter are eligible for autoclave sterilization.

If a standard sterile drape for the force gauge is also used, the SEAG can be used in sterile surgical procedures. A Bluetooth interface facilitates wireless in time transfer of the measured data to a personal computer for further data processing and storage.

The SEAG was attached to the testing station and was aligned with the tendon and muscle fibers of the supraspinatus.

### Measurement of Mobility

After grasping the distal tendon stump, the slide and hence the SEAG was lateralized stepwise, and the tendon stump was therefore displaced. Since the SEAG was aligned with the tendon and muscle fibers, longitudinal force was applied.

The digital force gauge allowed real-time measurement of the force loaded on the supraspinatus in Newton (Fig. 2a).

First, the tendon was preconditioned with ten cycles of 12 mm lateralization (1 mm/5 s). The tendon was then preloaded with 0.1 N to define the starting point (0 mm).

The measurement was then performed by lateralizing the tendon in 1 mm steps to a maximum of 12 mm. Preliminary testing had shown a risk of tendon slippage if a tendon lateralization higher than 12 mm was used. After each lateralization step (1 mm) and an equilibrium period of 5 s, the load in Newton was registered by the tensiometer. A test cycle was finished at 12 mm lateralization.

Using this approach, the mobility of the tendon was drawn in a force–displacement graph (N/mm).

Two different parameters were defined to describe tendon mobility in this study: *maximum load* on the tendon within 12 mm of lateralization and *stiffness* of the tendon, computed as the slope in the linear region of the force–displacement curve (10-12 mm).^39^ Both were tested for interobserver and intraobserver agreement.

*Obvious degenerative changes of the glenohumeral joint* (1), *preexisting defect of the rotator cuff* (2) and *damage of rotator cuff before definite measurements started* (3) lead to exclusion of the specimen. In the case of *tendon slippage within intraobserver agreement measurements* (4) and *duration of measurements *>* 2* *h postmortem* (5) the shoulders were excluded as well. Data from these shoulders were not used.

### Testing Protocol

#### Interobserver Agreement

Interobserver agreement testing was performed to evaluate the influence of attaching and detaching the grasper and the individual definition of the starting point. It is assumed that the actual measurement (after attachment of the grasper) as described above is very reliable because it is controlled mechanically by the test station. However, the moment of attaching the grasper and determining the starting point is vulnerable to ambiguity. Therefore two observers tested the interobserver agreement.

After preconditioning, the SEAG was detached from the tendon and the slide was brought in minimum lateralization (default position). Observer I then grasped the tendon and defined the starting point at 0.1 N. Then the first test cycle was carried out by observer I as described in the section “*measurement of mobility”.*

At the end of the test cycle, the SEAG was detached and the slide was brought to the default position. After grasping the tendon again and defining his own starting point at 0.1 N, observer II carried out his first test cycle in the same manner. Overall, both observers performed three test cycles in turn. Interobserver agreement was tested for maximum load and stiffness was calculated (Fig. 1).

#### Intraobserver Agreement

Intraobserver agreement was performed to evaluate the actual measurements. Therefore the SEAG was not detached between the test cycles.

The starting point was once again defined after preload, and the first test cycle was performed. After reaching the endpoint of 12 mm, the SEAG was not detached but brought back exactly 12 mm to the starting point. This was repeated three times without detaching the SEAG. Again agreement for maximum load and stiffness were calculated (Fig. 1).

### Force–Displacement Characteristics

Due to the lack of an existing gold standard for comparative validation, we decided to evaluate the force–displacement characteristics and tested the correlation between lateralization and load. Therefore data from all nine test cycles was used. The findings were compared to the few existing values about *in vivo* tension of human rotator cuff for comparative purposes. Change of mobility over time was also examined to exclude plastic deformation or rigor mortis.

### Statistical Analysis

Statistical analysis was performed by a biometrician from the Medical Faculty Mannheim of Heidelberg University, using the statistical software SAS, Release 9.4, (SAS Institute, Cary, NC, USA). Data have been entered in Microsoft® Excel for Mac Version 15.22 (Microsoft®, Redmond, USA) and then imported into SAS.

For quantitative variables, mean and standard deviation were calculated. For the assessment of the inter- and intraobserver reliability, the intraclass correlation (ICC) was calculated for every lateralization step based on a 2-way ANOVA.

Agreement strength was inferred from the ICC in accordance with the recommendations of Landis and Koch.^23^ ICC < 0.20 was interpreted as poor agreement, a value in the range of 0.21 to 0.40 as fair agreement, between 0.41 and 0.60 as moderate agreement, in the range 0.61 to 0.80 as substantial agreement, and above 0.81 as nearly perfect agreement.

The correlation between load and lateralization was calculated using Spearman’s correlation coefficient. Deviation of mobility over time was evaluated using an ANOVA for repeated measurements with *post hoc* testing according to Tukey–Kramer using the SAS procedure PROC MIXED. In general, a test result was considered statistically significant if the corresponding *p* value was less than 0.05.

## Results

The sample consisted of 7 left and 8 right shoulders. All prepared shoulders showed an intact rotator cuff without degenerative joint changes. Mean width of the tendon stump was 11.2 mm ± 2.3. Mean length of the myotendinous unit was 44.5 mm ± 3.5, which resulted in a mean relative lengthening of 27% ± 2 for maximal lateralization (Table 1). Tendon slippage was observed once and the shoulder concerned was excluded. For all included shoulders, the measurements were completed within 2 h postmortem.

**Table 1:** Tendon measurements and interclass correlation for inter- and intraobserver reliability.

	15 shoulders
Side	
Right	8
Left	7
Myotendinous length in mm	44.4 ± 3.4
Distal Tendon width in mm	11.4 ± 2.3
Rel. myotendinous lenghthening in %	27 ± 2

	Interobserver agreement	Intraobserver agreement
Maximal load (N)	29.8 ± 13.3	26.6 ± 11.6
Stiffness (N/mm)	5.1 ± 2.1	6.9 ± 3.1
Max. ICC	0.672	0.944

Categorical data as categorical data as frequencies and continuous data as mean ± standard deviation

The maximum load was found at 12 mm lateralization in all shoulders and test cycles and had a mean value of 28.6 N ± 12.5. The width of the tendon stump or length of the myotendinous unit did not affect the maximum load (*p* = 0.608; *p* = 0.972).

**Figure 3:**
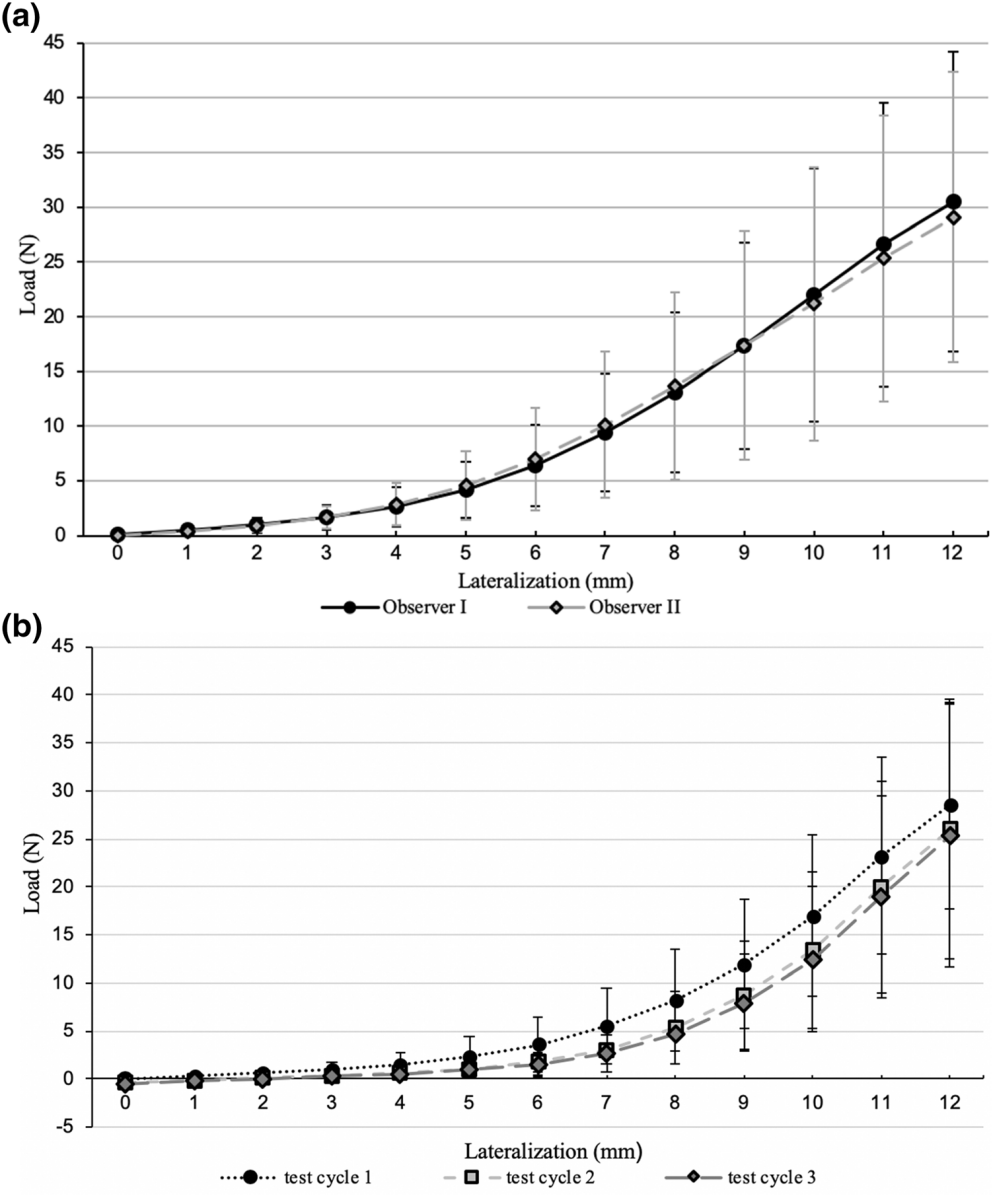
(a) Force–displacement graph for interobserver agreement measurements (in mean and ± for all shoulders) observer I vs. observer II. (b) Force–displacement graph for intraobserver agreement measurements (in mean and ± of all 15 shoulder) for 3 test cycles

In all test cycles, the load increased with progressing lateralization. It started with a non-linear part followed by a linear part (Figs. 3a and 3b). The Spearman’s correlation coefficient of 0.92 confirms the strong correlation between load and lateralization (*p* < 0.001). The mean calculated stiffness was 6.0 N/mm ± 2.6. There was no significant change over time for maximum load or calculated stiffness within 2 h postmortem (*p* = 0.427; *p* = 0.704).

### Interobserver Agreement

Interobserver agreement was strongly affected by the lateralization (Table 2). For load, the highest ICC values between both observers were found at maximum lateralization and demonstrated a substantial interobserver agreement (ICC 0.672). The interobserver agreement for calculated stiffness was 0.021 (slight agreement).

**Table 2:** Results of objectivity and reliability testing (intra-class correlation) for all 15 shoulders.

Lateralization (mm)	Interobserver agreement	Intraobserver agreement
1	0.316	0.206
2	0.287	0.162
3	0.325	0.159
4	0.331	0.230
5	0.414	0.363
6	0.491	0.520
7	0.578	0.594
8	0.635	0.684
9	0.622	0.771
10	0.657	0.849
11	0.672	0.914
12	0.624	0.944

### Intraobserver Agreement

For intraobserver agreement, a maximum ICC of 0.944 (“nearly perfect” agreement) was found (load). The reliability showed a strong association with the grade of lateralization (Table 2). Calculated stiffness showed an ICC of 0.774 (substantial agreement).

## Discussion

The aim of this study was to implement an animal model that provides biomechanical properties close to *in vivo* tissue for exploring the mobility of the supraspinatus myotendinous unit. A second aim was to introduce the SEAG, a novel device for tension measurement of the rotator cuff.

Our measurements reveal reproducible load–displacement curves for all tested shoulders. All shoulders exhibit nonlinear curves at the start of lateralization (toe-in region) followed by a linear section. This characteristic load–displacement correlation was previously reported for *in vitro* and *in vivo* tendons.^9^,^35^ The mean maximum load on the myotendinous unit was 28.6 N. This is quite similar to the intraoperative repair tension of *in vivo* human supraspinatus tendons (31.9 N^16^ 28.5 N^31^). The Spearman’s correlation coefficient confirms the strong correlation between load and lateralization (*p* < 0.001).

We could demonstrate substantial interobserver agreement and nearly perfect intraobserver agreement for the assessment of maximum load. Conversely, ICCs for calculated stiffness were quite low. ICC for the intraobserver agreement was 0.774 and for interobserver agreement 0.021. We found a mean calculated stiffness of 6.0 N/mm, which is much lower compared to existing data about stiffness of isolated tendons.^7^ Based on these findings, we cannot recommend using stiffness for mobility characterization.

Confounders such as plastic deformation of the tendon or effects of rigor mortis could be excluded.

In conclusion, we present a valid and reliable model for the clinically important but scientifically underrepresented topic of tendon mobility. We implemented a *in vivo*-close model by using fresh porcine shoulders within 2 h postmortem.

As it meets the design criteria of portability, sterilizability, and fast and intuitive handling in combination with high accuracy and reliable measurements of mobility, the SEAG is suitable for use in everyday clinical practice. Various shoulder models have been used in biomechanical studies.^6^ Porcine shoulders have been identified as a useful, cost-effective, and consistent model, and are widely used for research on rotator cuff surgery. Most of the studies investigated the insertion site after tendon reconstruction, especially bone-implant (type, number, and position of anchors) and implant-tendon interface (suture material, type, and number of knots). Parameters like load to failure or gap formation were the main focus.^13^,^29^ In these studies, the supraspinatus muscle was often dissected. Biomechanical properties, especially the mobility of the myotendinous unit, were neglected.

Some studies have elaborately engaged with the biomechanical properties of human rotator cuff tendon. Biomechanical, and often histological, parameters were analyzed using mostly small probes in fresh-frozen cadaver shoulders.^10^,^12^,^22^ This basic research accurately specified the biomechanical parameters of different parts of the tendon. However, this approach has limitations since the reaction of the entire muscle–tendon unit is far more complicated on loading.

For small animals, especially rats, biomechanical *in vivo* studies have tested the entire myotendinous complex.^27^,^30^ Soslovsky *et al*. implemented a murine model^35^ and tested the tendon mobility under several simulated conditions. They provided crucial knowledge about increasing repair tension over time following tendon injury, and the negative impact of high tension on rotator cuff repair *in vivo*.^8^,^9^ Unfortunately, despite its anatomic resemblance to the human shoulder, a rat model provides no easy transfer to human *in vivo* use due to the considerable difference in size and acting forces. Nevertheless, we have found similar characteristics of force–displacement curves compared to *in vivo* measurements of the murine supraspinatus.

Pastor *et al*. recently published a study about the impact of reposition technique on tendon tension, using frozen cadaver shoulders.^32^ In limitation, prior studies found altered biomechanical properties for frozen cadaver^2^ and the reported values for tendon tension differed substantially compared to *in vivo* findings.

Davidson *et al*. investigated mobility of the human rotator cuff *in vivo*.^4^ Using a rudimentary device (vicryl tag sutures fixed at the distal tendon stump and attached to a mechanical tensiometer outside the shoulder), they measured the tendon tension needed for rotator cuff repair. They showed a significantly better subjective and objective outcome for those reconstructions with low tendon mobility. Kim *et al*. showed an inverse correlation of repair tension with healing of reconstructed tendon.^16^ Recently Park *et al*. found a significant higher re-tear rate when tension > 35 N is required for reconstruction.^31^

The mobility of the torn tendon in surgical procedures has previously been addressed. Lo and Burkhardt reported favorable results after the arthroscopic interval slide procedure.^25^ Kim *et al*., however, found higher re-tear rates in patients after an aggressive interval release.^18^ They assumed an increased risk for devascularization with the additional posterior slide. Using the SEAG to verify if an appropriate level of mobilization has achieved might help here. Recently there has been much discussion about single vs. double-row refixation of the rotator cuff tendons.^3^,^11^,^40^ In this regard, a new re-tear type has been described.^20^ The “medial failure”, occurs proximal to the reconstruction side at the musculotendinous junction and might be caused by tendon strangulation and stress concentration. Consequently, the attention for a low tension repair is increasing. Double-row refixation has benefits in terms of tendon-bone contact, mechanical strength, and contact pressure. It demonstrates better anatomic healing in many studies. However, a medialized single-row refixation seems favorable for retracted tendons.^24^ Here the influence of tendon strangulation, reduced blood flow, and overtensioning is suspected. Choosing the appropriate surgical technique is difficult, as no absolute values regarding tolerance are available. The SEAG might therefore be beneficial for both scientific investigations and clinical practice.

The *in vivo* use of the method presented has considerable restrictions. The test station, which enables accurate lateralization, cannot be used *in vivo*. We assume that only the absolute tension on the tendon at complete reposition is relevant for *in vivo* use. This can easily be measured using the SEAG.

Human and porcine shoulders are very different. In particular, the porcine supraspinatus seems to have a different tendon–muscle ratio in favor of a pronounced muscle and a shorter tendon. However, published data regarding that observation were not found. In this study, we only created artificial supraspinatus defects by detaching the tendon from its footprint. This is most comparable to a non-retracted acute tendon rupture, which is quite rare in clinical practice. In fact, the majority of patients with rotator cuff tears have a retraction of the tendon, which is mostly due to the degenerative cause of a rotator cuff. Including shoulders with degenerative rotator cuff tears might lead to deviant results. Our measurements of tendon mobility were found to be quite similar to existing data for human *in vivo* supraspinatus tendons.

As this study aimed to test the reliability of the SEAG, a control group which allows a distinction between degenerative and acute rupture was not tested.^16^,^31^

It would be very interesting to compare tendon mobility characteristics of healthy and degenerative shoulders in future studies.

We performed the measurements under constant temperature (20 °C ± 0.5) to minimize the possible influence of rigor mortis. Under these conditions, we found no signs of rigor mortis within a measurement period of 2 h, which is consistent with previously published literature.^38^ Nevertheless, we found no discriminating biomechanical data about the impact of rigor mortis on larger animals, especially for the myotendinous unit of the supraspinatus.

With this feasibility study, we introduce an *in vivo*-like model for investigating rotator cuff tendon mobility. The mobility assessment of the myotendinous unit of fresh porcine shoulder gives a reliable measurement. With the SEAG, we have introduced a reliable device for the future *in vivo* assessment of tendon mobility in research and clinical settings.

## Abbreviations

SEAG: Sensor-enhanced, arthroscopic grasper
ICC: Intraclass correlation
